# Nutritional vitamin D deficiency: a case report

**DOI:** 10.1186/1757-1626-2-7000

**Published:** 2009-05-14

**Authors:** Rachel L Stevens, Corey Lyon

**Affiliations:** Research Family Medicine Residency, Research Medical Center6650 Troost Ave, Kansas City, 64131USA

## Abstract

We present a 6-month-old African American male child with a chief complaint of failure to appropriately gain weight despite adequate caloric intake via breastfeeding. While he has met developmental milestones he appears small for age and is diagnosed with failure to thrive after crossing two major growth curve percentiles. After appropriate diagnostic workup, a diagnosis of nutritional vitamin D deficiency (rickets) was reached and supplementation was initiated with ensuing adequate catch-up growth.

## Introduction

Rickets is often considered an “old” disease, a nutritional deficiency that has plagued communities for centuries. The re-emergence of vitamin D deficiency in westernized societies is thought to be multifactorial secondary to poor dietary intake, popularization of breastfeeding, and diminished exposure to sunlight. It is the most common metabolic bone disease in the world and is easily treatable as well as preventable with sun exposure and dietary supplementation [1].

Vitamin D is a prohormone essential for absorption of calcium from the intestines. Its supply stems from two well-known sources: exposure to sunlight and dietary intake, which accounts for less than 10% [2]. Vitamin D is primarily made in the skin after exposure to UV-B radiation (290 to 315 nm wavelengths) [2]. In rickets, decreased stores of this prohormone leads to low levels of ionized calcium, which initially stimulates parathyroid hormone release to initiate calcium resorption in the renal tubules (along with loss of phosphorous), and increase 1,25 dihydroxy vitamin D synthesis. A level of 25 hydroxy vitamin D (25-OH D) less than 12.5 nmol/L (5 ng/mL) is suggested for the diagnosis of rickets with a healthy maintenance level of approximately greater than 50 nmol/L (20 ng/mL) [1,2]. It should be noted that newer data suggests a lower limit of 80 nmol/L may be a more acceptable level in adults [1,2].

## Case presentation

We present a 6-month-old African American male child with poor interval growth. His mother has noticed that though he is thought to be breastfeeding appropriately, as defined by feeding 4 ounces of pumped breast milk every 2-3 hours, and has been meeting developmental milestones, his weight and height are not as expected. He has been exclusively breastfed and his mother has not introduced solid foods as of yet to his diet.

He was a full term, spontaneous vaginal delivery without complications during the pregnancy or labor. He was in the 50^th^ percentile for both height and weight at his 2 month visit, but has fallen to below the 3^rd^ percentile for weight and is at the 3^rd^ percentile for height. He is on no medications, there are no other siblings with failure to thrive and his mother has no post-partum depression or substance abuse issues. There is no family history of malabsorptive conditions. His mother and father are of normal stature.

His review of systems is negative for emesis, diarrhea, fever, appetite changes, swallowing abnormalities, respiratory symptoms, apnea, repeated acute illnesses, or frequent injuries.

His weight at the four month well child visit was 6477 grams with a length of 63.5 cm increasing to only 6761 grams and a length of 66 cm by his six month well child visit. His vitals signs are otherwise stable. His physical exam is significant for an alert, playful, developmentally appropriate child, small for his age. His head/neck, cardiac, respiratory, gastrointestinal, genitourinary, musculoskeletal and neurological exams were within normal limits.

He is appropriately diagnosed with failure to thrive at this visit based on deviation across two major percentiles on standardized growth curves. His estimated weight needs were calculated to 0.33 kg/month and a follow up visit was established in one month with addition of solid foods and continued breastfeeding with the addition of formula to pumped breast milk for increased caloric intake. His mother was also instructed to keep a strict food diary.

Interval weight gain was not maintained with a weight of 7045 grams at follow up despite adequate caloric intake estimated based on his food diary and formula supplementation. Laboratory studies ordered were complete metabolic profile, thyroid stimulating hormone (TSH), lead level, and complete blood count (CBC). Electrolytes, kidney function, bilirubin, AST, ALT, protein, albumin, TSH, CBC, and lead were all normal. Alkaline phosphatase was elevated at 4280 (on repeat 6310). Normal should be less than 500 IU/L in neonates and 1000 IU/L in children up to age 9. Follow-up labs including gamma glutamyl transferase (which was normal, suggestive of boney resorption) [2], C reactive protein, T3, free T4, phosphate, parathyroid hormone, and 25-OH D were ordered. Phosphate was low at 2.9 (normal 3.0-4.5) and the vitamin D level was 11 (45-50 ng/mL).

A skeletal survey was ordered showing metaphyseal fraying and cupping of bilateral distal femurs, bilateral proximal and distal tibiae and fibulae, bilateral proximal and distal humeri, bilateral distal radii and ulni and the distal aspects of 2^nd^ through the 5^th^ metacarpals most consistent with rickets of the extremities, see radiograph 1 and 2. These classic findings may be paired with a separation of the periosteum from the diaphysis secondary to unmineralized osteoid when evaluating radiographic evidence of rickets [1,3]. Radiographic improvement should manifest within 3 months of appropriate treatment. Underlying malabsorptive conditions or noncompliance should be considered if this is not observed [2].

**Figure 1:**
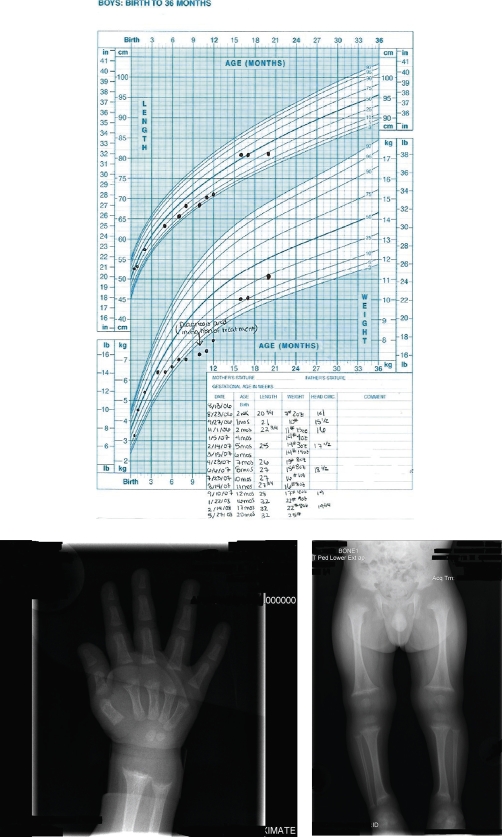
**Growth Chart:** Growth chart from birth to 20 months showing failure of appropriate growth and then his catch up growth after appropriate treatment. **Radiograph Wrist/Hand:** Metaphyseal fraying and cupping of distal radius and ulna and the distal aspects of the second through fifth metacarpals. **Skeletal survey:** Metaphyseal fraying and cupping involving bilateral distal femurs, bilateral proximal and distal tibiae and fibulae, bilateral proximal and distal humeri.

The diagnosis of rickets was made and the patient was started on 2000 IU of vitamin D and calcium carbonate 1000 mg daily. He was also started on iron sulfate 22 mg daily and Zinc 20 mg daily as recommended by pediatric endocrinology and nutrition staff. His catch-up growth has lead to a current weight (at 2 years of age) in the 45^th^ percentile and height in the 30^th^ percentile (Figure 1). Follow up labs (calcium, phosphorous, alkaline phosphatase) should be performed one month after therapy is initiated and again at 3 months along with magnesium, PTH, and 25-OH D [1,2]. Follow up labs in this patient indicated an improvement in vitamin D up to 29 ng/mL with ongoing supplementation continuing.

## Discussion

Infants and adolescents are predisposed to rickets secondary to increased flux in body composition and rates of rapid bone growth causing increased need/utilization of calcium and phosphate. Further increased risk is associated with dark-skinned individuals, lack of UV-B exposure, solely breastfed infants and prematurity. Screening should be considered for children with poor growth/development, seizure activity/tetany, and children with chronic malabsorptive states.

It is indisputable that breast milk is the ideal nutrition for infants, however, it only contains 15 - 50 IU/L of vitamin D [1,2,4]. There is limited prevalence estimates for vitamin D deficiency rickets in North America and the United Kingdom. Reported and published cases in the United States increased from 65 between 1975 to 1985 to 228 from 1986 to present [2]. This has led for the American Academy of Pediatrics (AAP) recommendation of vitamin D supplementation in all breast fed infants who do not consume 500 mL/day of vitamin D fortified formula and all non-breastfed infants that do not consume at least 500 mL/day of vitamin D fortified formula. It is recommended this begin in the first days of life and continue through childhood/adolescence [2]. The 2003 AAP breastfeeding guidelines suggest 200 IU/day to maintain a minimum level of 27.5 nmol/L, however this has been shown insufficient for prevention of all cases of rickets leading to controversy and newer recommendations for 400 IU/day, especially in deeply pigmented breastfed infants [2,5]. Despite appropriate breastfeeding technique and the appropriate amount of breast milk our patient was at risk for vitamin D deficiency secondary to the lack of oral vitamin D supplementation and his ethnicity.

The primary source of vitamin D (sunlight) is dependent on geographic location as well as outdoor exposure. To maintain a low normal level (>27.5 nmol/L) of vitamin D, a fully clothed child would have to spend two hours outside weekly and darker skinned individuals may require exposures up to 6-10 times this amount [1,2]. Sunscreen with an SPF of 15 reduces synthetic capacity by up to 98% [2,6]. The current AAP recommendation (to prevent sunburns and reduce skin cancer risk) is to keep infants less than 6 months of age out of direct sunlight and encourage the use of protective clothing/sunscreen again increasing the risk of vitamin D deficiency in this patient [2].

Because of this recommendation, management of vitamin D deficiency is via oral vitamin supplementation. Ergocalciferol (plant formulated vitamin D2) or Cholecalciferol (animal formulated vitamin D3) at >5000 IU daily for 2-4 months is suggested for toddlers greater than 12 months of age and up to 10,000 IU in adolescents. In younger infants (1-12 months) 1,000-5000 IU daily has been suggested. In infants <1 month old, 1000 IU daily is recommended [2]. Once laboratory values have normalized, maintenance of 400 IU per day is suggested [1,2]. If compliance is a concern a one time treatment with high dose oral formulation is appropriate (100,000-600,000 IU) over 1-5 days with a follow up dose in 3 months if necessary [2]. It is suggested that vitamin D3 may be up to 3 times as potent as D2 and may be preferable, especially when used in bolus form. Some formulations of vitamin D may contain propylene glycol, which is toxic at high doses so caution is advised [2].

## Conclusions

While rickets is a disease that has plagued society for many centuries it is certainly still a significant cause for both skeletal and non-skeletal complications in today's culture. Despite recommendations for supplementation in all breast-fed infants and fortification of multiple household food items, it is still a relatively common nutritional deficiency. Education on proper nutrition during pregnancy and supplementation during breastfeeding is necessary to prevent its growing resurgence. Proper childhood maintenance visits with growth and development screenings are critical for early detection of this easily treatable condition.

## List of abbreviations

AAP: American Academy of Pediatrics
ALT: Alanine Aminotransferase
AST: Aspartate Aminotransferase
CBC: Complete blood count
PTH: Parathyroid hormone
SPF: Sun Protection Factor
TSH: Thyroid stimulating hormone
UV-B: Ultra violet-B
